# Physicochemical characterization of sodium stearoyl lactylate (SSL), polyoxyethylene sorbitan monolaurate (Tween 20) and κ-carrageenan

**DOI:** 10.1016/j.dib.2018.05.064

**Published:** 2018-05-19

**Authors:** M.C. Ortiz-Tafoya, Alberto Tecante

**Affiliations:** Departamento de Alimentos y Biotecnología, Facultad de Química, Universidad Nacional Autónoma de México, Ciudad Universitaria, CdMx 04510, México

**Keywords:** Sodium stearoyl lactylate, Tween 20, κ-carrageenan, Surface tension, Calorimetry, IR, NMR

## Abstract

Surfactant-polymer mixtures are common in food, cosmetic and pharmaceutical products. These components can interact with each other. The interactions depend on the type of polymer and surfactant, the purity of the ingredients, the ionic content and their concentration. Therefore, the data presented here provide valuable information that could be useful for those working with these mixtures in different applications, particularly in blends with polyelectrolytes and their counterions. This article contains experimental data about the physicochemical characterization of sodium stearoyl lactylate (SSL), polyoxyethylene sorbitan monolaurate (Tween 20) and κ-carrageenan. Techniques included atomic absorption, DSC, FTIR-ATR, NMR, and surface tension.

**Specifications Table**

**Table:** 

Subject area	*Chemistry*
More specific subject area	*Physical chemistry*
Type of data	*Tables and figures*
How data was acquired	*Atomic absorption spectrometer 3110 (Perkin Elmer); Spectrophotometer with an ATR universal accessory (Spectrum 400, Perkin Elmer Cetus Instruments); Microcalorimeter μDSC 7 Evo (Setaram); Microtensiometer EZPiplus (Kibron) and spectrometer AVANCE-III 500 (Bruker)*
Data format	*Analyzed results*
Experimental factors	*Commercial forms were used without further purification*
Experimental features	*Physicochemical properties were determined for κ-carrageenan and two surfactants (SSL and Tween 20) by using atomic absorption, infrared and nuclear magnetic resonance, calorimetry, and measurements of surface tension*
Data source location	*Authors’ affiliation*
Data accessibility	*Data are presented in this article*

**Value of the data**

• Intrinsic ions content is a key factor in mixtures of ionic components, like κ-carrageenan and SSL, because interactions between them can be affected by the presence of salts.
• DSC analysis, IR and NMR spectra are used to identify the components and to determine their purity, which are relevant characteristics since the presence of distinct species could modify their behavior.
• Surface tension is useful to know the adsorption parameter of surfactants and how it changes in a mixture with other components.

## 1 Data

SSL had sodium as the primary ion and small quantities of other ions, whereas Tween 20 only had traces of ions. κ-carrageenan was found mainly as the potassium salt form since it is the primary ion. The IR spectrum of SSL showed typical carbonyl, ether groups, and aliphatic linear chain bands. Tween 20 showed an OH band, and characteristic carbonyl and ether groups bands. The spectrum of κ-carrageenan showed sulphate ester and glycosidic linkage bands, which are not due to the presence of ι-carrageenan because the NMR spectra show a kappa: iota hydrogen molar ratio of 1:0.073. The melting transition temperature of SSL was 54.6 °C and the recrystallization temperature 40.5 °C. Tween 20 did not show any apparent change over the working temperature range. The thermal behavior of κ-carrageenan depends on the ionic content since its gelation is affected by the presence of K^+^ ions. Therefore, the addition of KCl increased the transition temperature and enthalpy compared with κ-carrageenan without the salt. The addition of KCl modified the critical micelle concentration (CMC) of SSL solutions because of the chemical nature of the surfactant. This effect was not observed for Tween 20. The CMC values were higher for SSL due to electrostatic repulsion between its head groups. The same behavior was observed in mixtures of κ-carrageenan with the individual surfactants.

## 2 Experimental design, materials and methods

### 2.1 Materials

Materials included food-grade κ-carrageenan (Ingredients Solutions, USA) without further purification, powder sodium stearoyl lactylate (Palsgaard, Juelsminde, Denmark), liquid Tween 20 (Hycel de México S.A. de C.V., Mexico), and potassium chloride (Merck, Germany). Solutions were prepared in deionized water; its resistivity was greater than 50 kΩ·m and its total organic carbon (TOC), less than 30 ppb.

### 2.2 Atomic absorption

The ion concentration of SSL, Tween 20 and κ-carrageenan (Table 1) was determined in an atomic absorption spectrometer 3110 (Perkin Elmer, USA) equipped with a deuterium background corrector, using an air-acetylene flame. Wavelengths employed to identify Na^+^, K^+^, Ca^2+^, and Mg^2+^ were 330, 404, 422 and 202 nm, respectively. All samples were previously digested with a mixture 80:20 of nitric to sulfuric acids at 90 °C for 2 h or until the disappearance of the combustion gas of the raw material.

**Table 1:** Intrinsic ion content (mg·kg^−1^) of κ-carrageenan, SSL and Tween 20.

Ion	κ-carrageenan	SSL	Tween 20
Na^+^	21,514 ± 1727	21,700 ± 460	1 000 ± 30
K^+^	62,100 ± 1684	1 200 ± 170	1 100 ± 10
Ca^2+^	1301 ± 61	100 ± 10	500 ± 10
Mg^2+^	< 10	< 10	100 ± 10

### 2.3 Fourier transform infrared-attenuated total reflectance (FTIR-ATR)

**Fig. 1:**
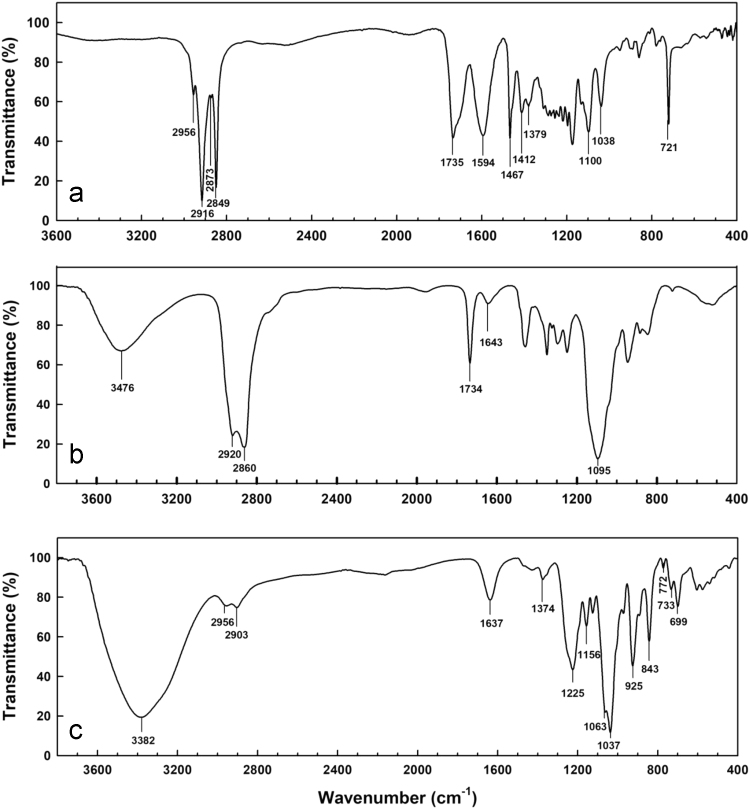
FTIR-ATR spectra of (a) sodium stearoyl lactylate, (b) Tween 20 and c) κ-carrageenan.

FTIR-ATR tests were performed in a spectrophotometer with an ATR universal accessory (Spectrum 400, Perkin Elmer Cetus Instruments, USA). The spectra were collected in the range of 4000–400 cm^−1^ with a resolution of 4 cm^−1^ with 32 scans per sample. Fig. 1 shows the IR spectra for SSL, Tween 20 and κ-carrageenan. Table 2 shows their characteristic absorption bands, allocated according to available sources [1], [2], [3], [4], [5].

**Table 2:** Major absorption bands of SSL, Tween 20 and κ-carrageenan IR spectra.

Component	Absorption (cm^−1^)	Functional group
SSL	2956/2873	Asymmetric and symmetric methyl and methylene stretch vibrations.
	2916/2849	
	1735	Carbonyl stretching from R-CO-O-R
	1594	Carbonyl stretching from R-CO_2_^−^
	1467/1379/721	Methylene/methyl band, methyl band, methylene rocking vibration. Evidence of a long aliphatic linear chain
	1412	Methyl symmetric bend vibration
	1100/1038	C-O stretching vibration
Tween 20	3476	Hydrogen-bonded O-H stretching
	2920/2860	Asymmetric and symmetric methylene stretching vibrations
	1734	Carbonyl group from R-CO-O-R
	1640	Carbonyl stretching
	1095	Stretch vibration of -CH_2_-0-CH_2_-
κ-carrageenan	3382	Hydrogen-bonded O-H stretching
	2956/2903	Methine stretch vibrations
	1640	Polymer bound water
	1374	Sulphate stretching vibration
	1225	Ester sulphate group asymmetric stretching
	1156	C-O-C asymmetric stretching
	1063/1037	C-O and C-OH modes and glycosidic linkage
	925	C-O stretching vibrations of 3,6-anhydro-D-galactose
	843	D-Galactose-4-sulphate
	772/733/699	Skeleton bending of pyranose ring

### 2.4 Nuclear magnetic resonance (NMR)

**Fig. 2:**
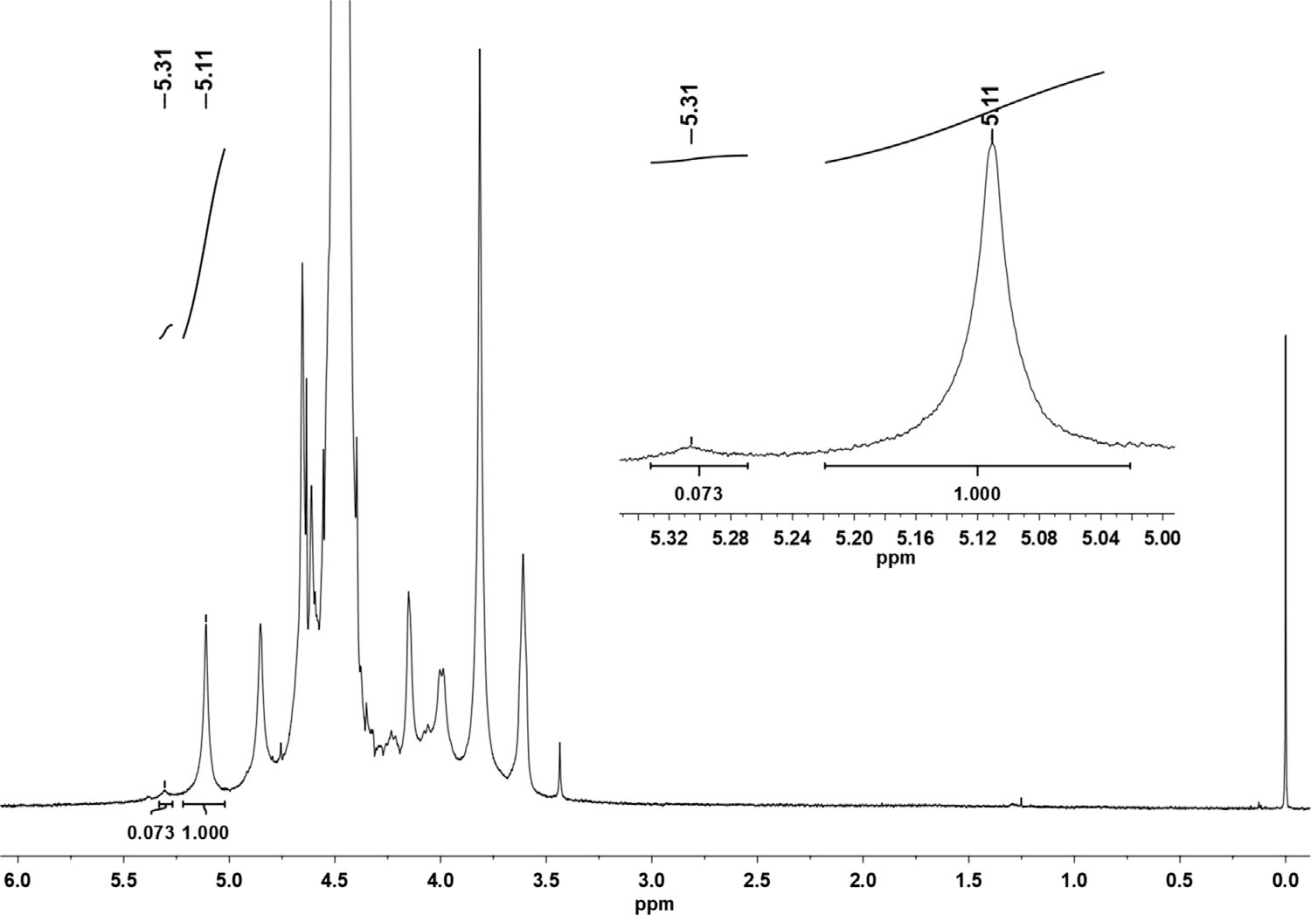
^1^H NMR spectra of κ-carrageenan. The signal at 5.11 ppm is due to the α-anomeric proton of κ-carrageenan, while the signal at 5.31 is for ι-carrageenan [6]. The internal reference (TSP) is at 0 ppm.

^1^H NMR spectra of κ-carrageenan (Fig. 2) were obtained with a spectrometer (AVANCE-III 500, Bruker, USA), operated at a frequency of 500 MHz. Tests were carried out using a BBI-5 mm with z-field gradient at 40 °C and 3-(trimethylsilyl)-2,2,3,3-tetradeuteropropionic acid (TSP) was added as an internal reference. A mass of 5 mg of κ-carrageenan was added to D_2_O, to a final concentration of 0.5%, and the dispersion was heated to dissolve the polysaccharide. This solution was used for NMR analysis. Data were processed with a commercial software (The MestReNova, v12.0.0-20080, Spain). The α-anomeric protons for κ and ι-carrageenan where identified from reported data [6].

### 2.5 Differential scanning calorimetry

**Fig. 3:**
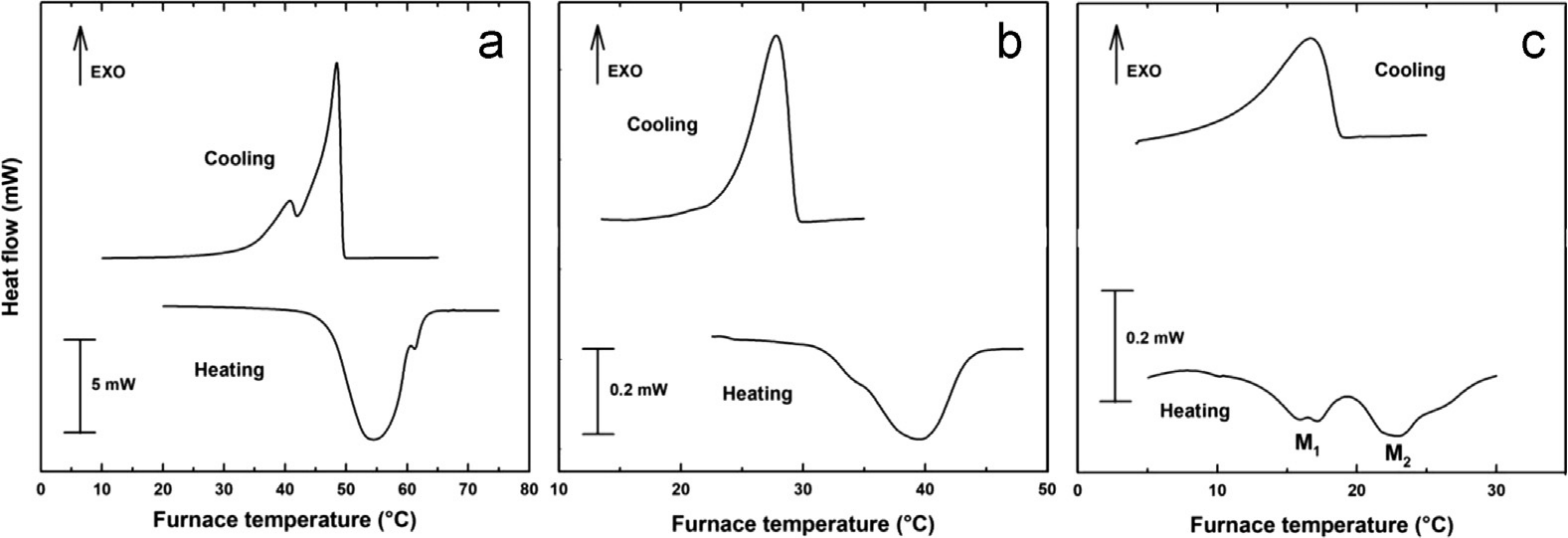
DSC heating and cooling thermograms for (a) sodium stearoyl lactylate at a rate of 1.2 °C min^−1^, (b) 0.5% κ-carrageenan + 13 mM KCl at a rate of 0.8 °C min^-1^ and (c) 0.5% κ-carrageenan at a rate of 0.8 °C min^−1^. M_1_ = Melting 1 and M_2_ = Melting 2.

DSC analysis (Fig. 3) was carried out in a microcalorimeter (μDSC 7 Evo, Setaram, France) under N_2_ gas atmosphere. SSL was analyzed at a rate of 1.2 °C·min^−1^ from 2 to 80 °C in one heating-cooling cycle using 30 mg of a powder sample. The reference cell was empty. κ-carrageenan was analyzed using a solution with 0.5% with or without 13 mmol·dm^−3^ KCl, at a rate of 0.8 °C·min^−1^ from 2 to 80 °C in three heating-cooling cycles using 600 mg of sample. The reference cell contained deionized water with or without 13 mmol·dm^−3^ KCl. Calorimetry data were analyzed with commercial software (OriginPro 2016, b9.3.226, USA). Enthalpies were expressed in J·g^−1^ of dry matter. Transition temperatures and enthalpies for SSL and κ-carrageenan are shown in Table 3.

**Table 3:** Transition temperatures and enthalpies for SSL and κ-carrageenan in the presence and absence of KCl.

Component		Temperature (°C)	Enthalpy (J·g^−1^)
SSL	Melting	54.6 ± 0.01	124 ± 0.15
	Gelling	40.5 ± 0.02	101 ± 0.17
κ-carrageenan	Melting 1	15.88 ± 0.02	5.46 ± 0.17
	Melting 2	23.02 ± 0.01	10.10 ± 0.07
	Gelling	16.72 ± 0.01	19.28 ± 0.09
κ-carrageenan + 13 mmol·dm^−3^ KCl	Melting	39.82 ± 0.06	36.08 ± 0.29
	Gelling	28.02 ± 0.02	33.92 ± 0.37

### 2.6 Surface tension

Surface tension was measured at 25 ± 0.5 °C with a microtensiometer (Kibron EZPiplus, Finland). The instrument was calibrated with deionized water, and 3 mL of sample was used for each test. Samples of SSL were prepared from dilutions of stock solutions. The latter were prepared by dissolution of the surfactant in hot water (60 °C); KCl was added when it was necessary. Samples of Tween 20 were prepared as SSL samples, but without heating. Samples of surfactant-κ-carrageenan were prepared by mixing surfactant and κ-carrageenan stock solutions with deionized water, after the combination of both components the system was heating at 92 °C to dissolve κ-carrageenan. Surface tension was measured 18 h after solutions preparation to let them to stabilize.

**Fig. 4:**
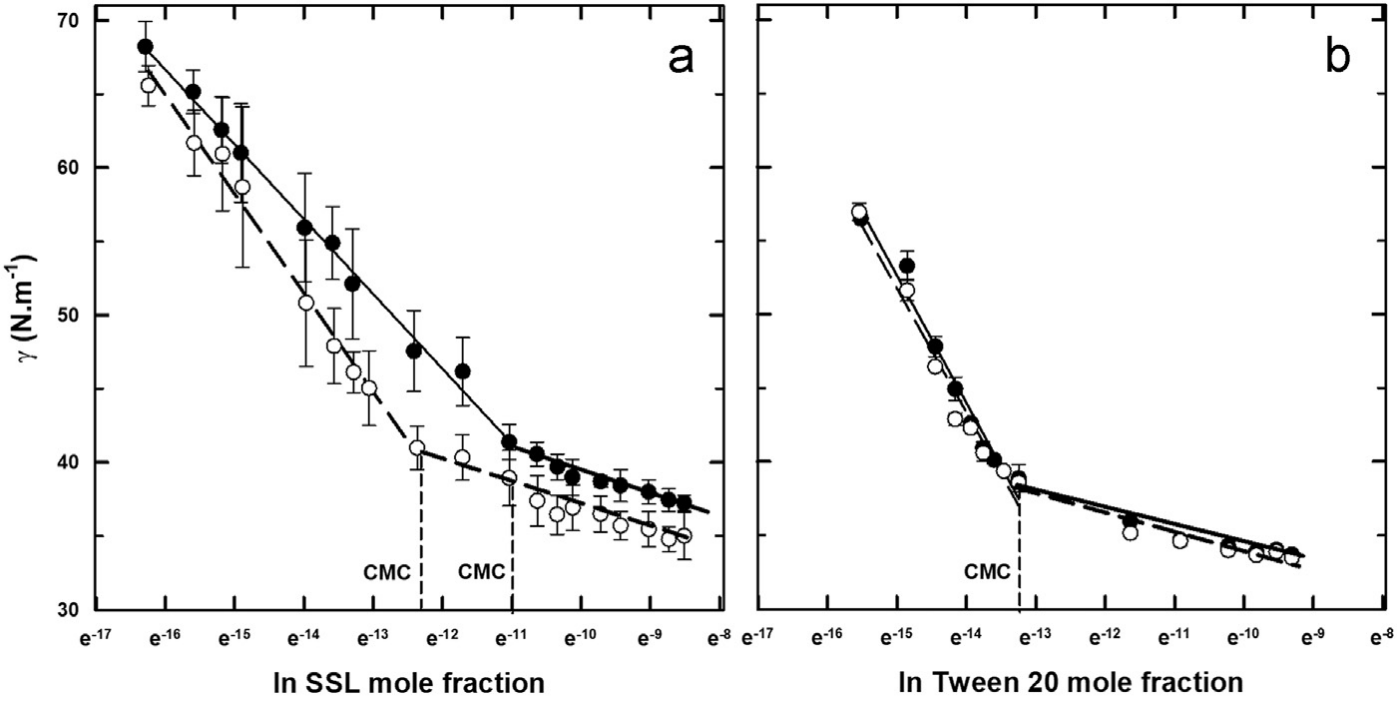
Influence of (a) sodium stearoyl lactylate and (b) Tween 20 concentration on the surface tension of water without (full circles) and with KCl (empty circles) at 25 °C. CMC = Critical micelle concentration.

The CMC was obtained from the variation of surface tension with surfactant concentration as shown in Fig. 4. The excess surface concentration, Γ, at constant pressure and temperature, was calculated from the Gibbs adsorption isotherm (Eq. 1) for surfactant and surfactant + KCl solutions [7].

(1) $$\Gamma_{\left(T,P\right)}=-(d\sigma /dln\;x_{s})/nRT$$

where R is the gas constant (8.314 J·K^−1^·mol^−1^), T absolute temperature (298 K), σ surface tension (N·m^−1^), x_s_ mole fraction of surfactant; and n is the number of independent components. It was used a molar mass of 451.6 and 1227.5 g·mol^−1^ for SSL and Tween 20, respectively, to calculate the fraction mol. For nonionic surfactants, neutral molecules or ionic surfactants in the presence of excess electrolyte n = 1 and for 1:1 ionic surfactants, assuming electrical neutrality of the interface, n = 2. From the excess surface concentration, the area per molecule, A, was calculated according to Eq. 2.

(2) $$A=1/\Gamma_{\left(T,P\right)}N$$

where N is Avogrado´s number; 6.022·10^23^ mol^−1^ [8]. The standard free energy of micellization was determined using Eq. 3 for the nonionic surfactant, and Eq. 4 for the ionic surfactant [9]. The information obtained from Eqs. (1), (2), (3) is summarized in Table 4.

(3) $$\Delta G_{CMC}=RT\cdot ln\left(x_{CMC}\right)$$

(4) $$\Delta G_{CMC}=(2-\beta )RT\cdot ln\left(x_{CMC}\right)$$

where, β is the counter-ion dissociation constant and x_CMC_ is the surfactant mole fraction at the CMC. According with Tandros [10], the degree of dissociation for many ionic surfactants are ~ 0.2, so Eq. 4 is as follows:

(5) $$\Delta G_{CMC}=1.8RT\cdot ln\left(x_{CMC}\right)$$

**Table 4:** Critical micelle concentration (CMC), excess surface concentration (Γ), molecular area (A) and free energy of micellization of SSL and Tween 20 without and with KCl calculated from their mole fraction.

	KCl (mmol dm^−3^)	CMC × 10^6^ (*X*_S_)	Γ·10^6^ (mmol·m^−2^)	A (nm^2^)	ΔG_CMC_ (kJ·mol^−1^)
SSL	0	16.1	1.023	1.624	-49.25
	13	4.26	2.692	0.617	-55-18
Tween 20	0	1.77	3.487	0.476	-32.83
	13	1.77	3.384	0.491	-32.84

**Fig. 5:**
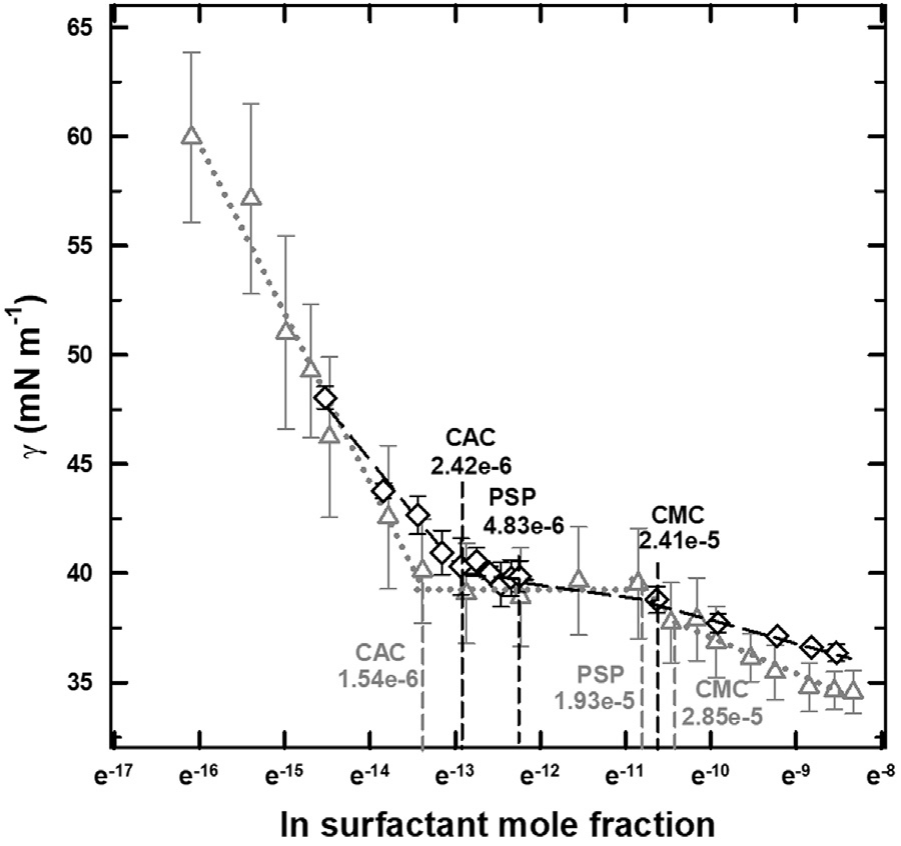
Influence of sodium stearoyl lactylate (gray triangles) and Tween 20 (black diamonds) concentration on the surface tension of 0.5% of κ-carrageenan solutions at 25 °C. CAC = Critical aggregation concentration, PSP = Polymer saturation point, CMC = Critical micelle concentration. Units are mmol·dm^−3^.

Data obtained for mixtures of κ-carrageenan with SSL and Tween 20, are presented in Fig. 5. Adsorption parameters (Γ, Α and ΔG) were not calculated, since the combinations of κ-carrageenan with the surfactants presented three break points in the graph. The first point is the critical aggregation concentration (CAC), and corresponds to the concentration where interaction between polymer and surfactant occurs. The second is known as the polymer saturation point (PSP) and represents the surfactant concentration at which the polymer-surfactant complex is desorbed from the interface. Finally, the third point occurs when the surface tension of the polymer-surfactant complex reaches the surface tension of the surfactant solution [11].

The CMC value, 0.05 wt%, for SSL was different from those, 0.08–0.10 wt%, reported by others [12]. This discrepancy can be explained by impurities present in the commercial sample. Another factor could be the SSL1 to SSL2 proportion, both molecules are the main components of the sample, and have different interfacial activity [13].

### 2.7 Statistical analysis

Means and standard deviations were calculated with Microsoft® Office Excel® 2007.
