# Supplementary material for: Physicochemical characterization of sodium stearoyl lactylate (SSL), polyoxyethylene sorbitan monolaurate (Tween 20) and κ-carrageenan
Source: Data Brief. 2018 May 19;19:642–50. doi: 10.1016/j.dib.2018.05.064 (PMC5997900; doi:10.1016/j.dib.2018.05.064)
Supplement: Supplementary file 1 — Supplementary material [file mmc1.doc]

UNIVERSIDAD NACIONAL AUTÓNOMA DE MÉXICO

DEPARTAMENTO DE ALIMENTOS Y BIOTECNOLOGÍA

FACULTAD DE QUÍMICA “E”

CD. UNIVERSITARIA, MÉXICO, CdMx, 04510

tel. +52-55-5622-5307; Fax +52-55-5622-5309

e-mail: [tecante@unam.mx](mailto:tecante@unam.mx),

April 25, 2018

Managing editor

Data in Brief

Dear Managing Editor,

We submit the second revised version, DIB-D-17-01377R2, of the manuscript “Physicochemical characterization of sodium stearoyl lactylate (SSL), polyoxyethylene sorbitan monolaurate (Tween 20) and κ-carrageenan” by María del Carmen Ortiz-Tafoya and Alberto Tecante, following the comments of the reviewers. Also, we provide in a separate document our responses to the reviewers’ observations and an Excel file with raw data as Supplementary Material.

We have read and agreed with the content of the revised manuscript and attest to the validity and legitimacy of the changes made. We declare no conflict of interest.

We hope the revised version will satisfy the standards of Data in Brief.

Sincerely,

Alberto Tecante
